# Chorda Dorsalis System as a Paragon for Soft Medical Robots to Design Echocardiography Probes with a New SOM-Based Steering Control

**DOI:** 10.3390/biomimetics9040199

**Published:** 2024-03-27

**Authors:** Mostafa Sayahkarajy, Hartmut Witte, Ahmad Athif Mohd Faudzi

**Affiliations:** 1 Fachgebiet Biomechatronik, Technische Universität Ilmenau, 98693 Ilmenau, Germany; 2 Centre for Artificial Intelligence and Robotics, Universiti Teknologi Malaysia, Kuala Lumpur 54100, Malaysia; athif@utm.my

**Keywords:** soft robotics, biomimetic development, self-organizing map, robot-assisted echocardiography

## Abstract

Continuum robots play the role of end effectors in various surgical robots and endoscopic devices. While soft continuum robots (SCRs) have proven advantages such as safety and compliance, more research and development are required to enhance their capability for specific medical scenarios. This research aims at designing a soft robot, considering the concepts of geometric and kinematic similarities. The chosen application is a semi-invasive medical application known as transesophageal echocardiography (TEE). The feasibility of fabrication of a soft endoscopic device derived from the *Chorda dorsalis* paragon was shown empirically by producing a three-segment pneumatic SCR. The main novelties include bioinspired design, modeling, and a navigation control strategy presented as a novel algorithm to maintain a kinematic similarity between the soft robot and the rigid counterpart. The kinematic model was derived based on the method of transformation matrices, and an algorithm based on a self-organizing map (SOM) network was developed and applied to realize kinematic similarity. The simulation results indicate that the control method forces the soft robot tip to follow the path of the rigid probe within the prescribed distance error (5 mm). The solution provides a soft robot that can surrogate and succeed the traditional rigid counterpart owing to size, workspace, and kinematics.

## 1. Introduction

Through learning about the efficiency and adaptability of organisms, bioinspired soft robots gain growing research interest. “Soft”, “flexible”, and “compliant” roughly correlate but address different properties. In conventional manipulators, flexibility can be leveraged to protect people in human–robot interaction or to minimize energy consumption [1]. Such robots rely on compliance due to the slenderness of their links or rotational spring effects implemented in the joints. “Soft robotics”, on the other hand, has so far found little acceptance in commercial practice. The main obstacles are doubts about the precision of production, but also movement precision and control complexity. With this paper, we would like to contribute a bionic approach to the utilization of soft robotics principles in products. To this end, we have focused on a particularly critical field of application for biomechatronics and biomedical engineering.

In the more challenging task of how established biomedical engineering systems can be improved biomimetically with compliant mechanisms, endoscopic systems are currently one of our study objects under consideration. Continuum robots (which are not really using continua) inspired by creatures like snakes or by elephant trunks have the ability to pass through narrow orifices and access points [2]; for example, inside the human body, or for observation purposes, via endoscopy, or operations, or in surgical robots [3,4]. Conventional medical probes and transoral robotic mechanisms contain hard components (either rigid or elastic, serial or parallel links [5]), and lack the softness and thus adaptability of natural organisms [6,7]. The noncompliant material of such mechanisms introduces high-risk hazards in human–machine interaction by applying unwantedly high mechanical forces on the tissue. This hazard can be seriously critical in the case of invasive or semi-invasive medical applications, where an end effector is manipulated inside the patient’s body. The current technology of rigid mechanisms employs joints with bearings, gears, and different types of coupling. The metal parts make the system heavy and provoke additional problems due to friction and rigidity.

For the mesoscale range of medical devices like endoscopic devices, we see the opportunity for an improvement in the performance by an approach based on biomimetics. We tested whether a biomimetic approach may improve performance and decrease security risks. For this test, a demonstrator system was developed. The proposed system can be described as a soft continuum robot. The kinematics of continuum robots can be described with transformation matrices [2] efficiently. However, especially for novice users, their manipulation and navigation have complexities, and researchers investigate artificial intelligence (AI) to enhance the manipulation dexterity [8]. 

For the technical part of biomimetics, we chose the improvement of the transesophageal echocardiography (TEE) probe. TEE is a cardiac imaging modality used to obtain cross-sectional images of the patient’s heart based on backscattered ultrasound (US) waves [9]. Measurements are taken from the direct neighborhood of the heart, thus avoiding the masking effects of the rips in external echocardiography and allowing complete 3D reconstruction of the organ. A conventional TEE probe carries a US transducer mounted at the tip of an endoscope, which is manually operated by a physician. The TEE process underlies significant risks such as human errors, exposure to ionizing radiation, and multitasking complexity, leading to injuries. While medical devices are fabricated with the highest standards, their possible applications are restricted by technological limitations. Further improvements in terms of control, safety, and workload of the operator must be found beyond the established design and fabrication methods of commercialized industrial systems. 

The traditional endoscopic probes used in invasive or semi-invasive modalities such as TEE and gastroenterology use an insertion tube to convey the sensor into the patient’s body. Then, the concept is to orientate the rigid “head” of the endoscope by bending the connection (“neck”) to the following semi-rigid body in two DoFs in such a way that driven by axial forces, the head glides through the anatomically preformed cavities with minimized contact, and if it occurs, minimized contact forces. The TEE probe can be explained as a wire-driven linkage of rigid parts, providing mechanical bending in the right–left and up–down directions. The TEE tube diameter (approximately 1 cm) is not convenient to pass through the human esophagus. Although the insertion part is termed flexible, it is rather stiff, and when it is bent during the operation, the bent section is not compliant. The relative motion between the head and tail may be described around one bending axis defined by the overlay of the two bending components. A critical hazard in the TEE operation is that the physician fails to straighten the probe before removing the probe from the esophagus. Manipulation of the probe in an incorrect position in the patient’s body exerts high forces that can rupture the soft tissues. In addition to hazards and difficulties threatening the patients, the traditional probes have limitations for the users (i.e., the sonographers) in terms of manipulation and ergonomics. It is difficult and needs intense training to manipulate the probe, especially using a single hand. Turning the knobs needs moderate finger force, and tip position control is not easy. 

Assurance of the quality of the application process counts on the training of the users and their skills. Researchers thus propose robot-assisted solutions for ultrasonic examinations [10,11]. Some robots have been developed to assist TEE [12,13,14] that are actually master–slave holders, manipulating the probe handle for the remote user. In [14], it is discussed that such holders have no control over the subsection of the probe between the tip and holder, and an additional support arm was suggested to alleviate the problem. Such rigid robots keep the probe without any change and add further complexities to it, while a soft robot may serve as a replacement for the probe. Soft robotics, employing the concept of biology for engineering (Bio4Eng [15]), mimics the soft body of live creatures or replicates biological systems [16,17]. Soft actuators, powered by pneumatic or fluidic pressures, are concurrently developed to replicate biological muscles [4,18,19]. They are, essentially, of low weight and do not need bearings, gears, and metal parts, and thus avoid the aforementioned problems. Applicability of existing control equipment like pneumatic valves is an advantage of soft robots that work with industrial-level pressures, although research is ongoing to develop fully soft pneumatic logic systems [20,21,22]. Soft robotics is seen as a new way to redesign future generations of current medical systems, attracting investors and companies for product commercialization. The vision is not limited only to the replication of conventional systems and competing with them but tackling a set of problems that existing technologies have not been able to solve. In this perspective, biomedical applications of soft robotics embrace rehabilitation, tissue engineering, soft biological cell biology, flexible surgical manipulators, etc. [4]. In particular, continuum robots that play the main roles in many robotic surgery and US scenarios can be supplemented by soft robots. 

Two main gaps are identified between pneumatic SCRs and the clinical application. The first is that the feasibility of fabrication and capability of soft robots for the defined intended medical application has not been investigated. The second is related to the usability of SCRs considering that the sonographers would not exhibit their dexterity when the traditional system is replaced with a new robot that is essentially of different mechanisms and DoFs. To address the gaps, two main goals were considered for this research. The first target is to design and prototype a pneumatic SCR with the size of the conventional probe and investigate the capability of the SCR to capture required bending and gestures. The second target is to develop an AI kinematic matching algorithm that basically projects the workspace of the SCR on that of the conventional system. 

This paper is organized as follows. Section 2 starts with a subsection introducing the conventional TEE probe and the equations and relations describing its kinematics. Next, a subsection is devoted to revisiting the system from biomimetics and transitioning to a soft conceptual design. The third subsection describes the realized SCR, the proposed model, and a novel self-organizing map (SOM)-based algorithm developed for controlling the robot. Section 3 explains the experimental and simulation results. First, the demonstrator is tested experimentally as a verification of the abilities of the SCR. Then, the next subsection represents the kinematic matching results for the whole workspace and some typical trajectories. Discussions of the outcomes, future directions, and conclusions are given in the last two sections.

## 2. Materials and Methods

### 2.1. Existing Reference System

**Figure 1:**
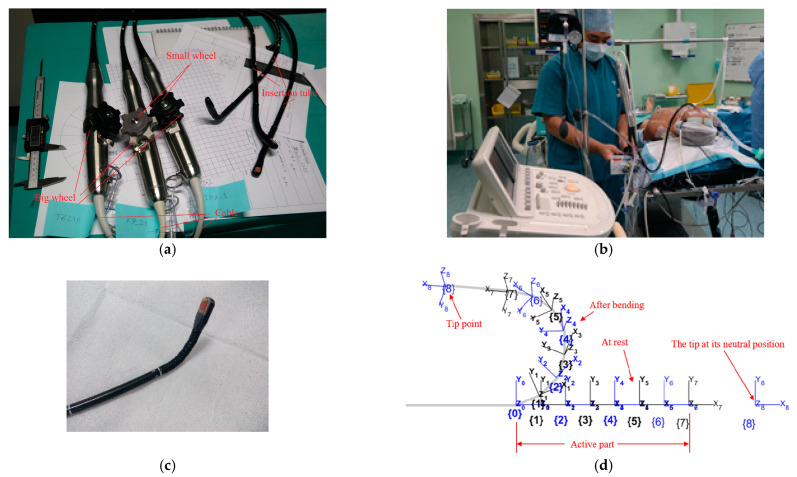
The conventional system: (**a**) Philips^®^ TEE probes used for adult patients; (**b**) example of application in surgery; (**c**) the distal section; (**d**) linkage coordinate systems. (Photos were taken by the authors during a hospital attachment.)

The TEE probe mechanism can technically be described as serially connected rigid links driven by a cable passed through the links. The cable goes around a pulley and is driven manually with rotation knobs known as “big” and “small” wheels. Figure 1a shows actual probes used in hospitals for adult patients. The probes are of different types, namely T6210, X7-2t, and IPX-1 from Philips^®^. All probes possess the same mechanical dimensions and exhibit similar bending when the wheels are rotated. This rotation causes the straight part at the tip to be located in the desired pose. The fact that only the distal section of the probe is inserted in the patient’s body, as in Figure 1b, encourages us to think of a soft robot as a replacement. The distal section is shown in Figure 1c. The proximal part of the tube, from the active part to the handle, is mechanically passive. The big wheel provides the anteflexion–retroflexion, and the small wheel provides lateral bending. 

The probe kinematic model can be constructed of two parts, where the first part maps the inputs, which is the rotation of the wheels of the TEE probe [14] to the probe states (bending and flexing angles). The same pattern is used for the soft probe where the inputs (contraction of the McKibben actuators) are mapped to the segmental bending and rotations. The second part of the kinematic model relates the states to the tip position. The state–output relations can be used for workspace evaluation for the different probes. For the TEE probe, a model is considered to consist of some serially connected links as in Figure 1d, where X_8_Y_8_Z_8_ is located at the tip and Z_8_ is normal to the sensor. Each link rotates around the Z-axis of the previous link in anteflex–retroflex, and around the relevant Y-axis in the lateral bending. Mathematically, each coordinate *i* is obtained by rotating the previous system of angle $\theta_{zi}$ around Z_i−1_, followed by a translation *l_i_* (=length of the link) along the new coordinate, followed by a rotation $\theta_{yi}$ around the new Y-axis as follows:

(1) $$T_{i}=Rotz(\theta_{zi})Trans(l_{i},0,0)Roty(\theta_{yi})$$

Ignoring friction and extensibility of the cables, it can be supposed that the links have similar conditions, and so the links show equal rotations, i.e., $\theta_{zi}=\theta_{z}$, $\theta_{yi}=\theta_{y}$. A homogeneous transformation matrix, *H*, relates the tip position in frame 8 to its position vector in frame 0 as

(2) $$H=T_{1}T_{2}T_{3}\ldots T_{8}\begin{array}{ccc} & , & \end{array}{\vec{P}}_{Tip}^{0}=H{\vec{P}}_{Tip}^{8}$$

The reachable workspace is defined as the set of tip point positions, ${\vec{P}}_{Tip}^{0}$, that can be reached by some choice of the state vector, ${\vec{q}}_{R}$, within the set of admissible states $Q^{rigid}$

(3) $$W^{R}=\left\{\left.{\vec{P}}_{Tip}^{0}\right|{\vec{q}}_{R}\in Q^{rigid}\right\}\subset {\mathbb{R}}^{3}$$

where the state vector is described by the constant curvature rule as ${\vec{q}}_{R}={\left[\theta_{z},\theta_{y}\right]}^{T}$; and $Q^{rigid}$ can be represented by the range of the angles obtained from measurements as

(4) $$Q^{rigid}=\left\{\vec{q}\left|0<\theta_{z}<18{}^{\circ},0<\theta_{y}<7{}^{\circ}\right.\right\}$$

Then, using Equations (2) and (4) in Equation (3), a set of reachable points is obtained, denoted as *W^R^*.

### 2.2. Biomimetic Concept

**Figure 2:**
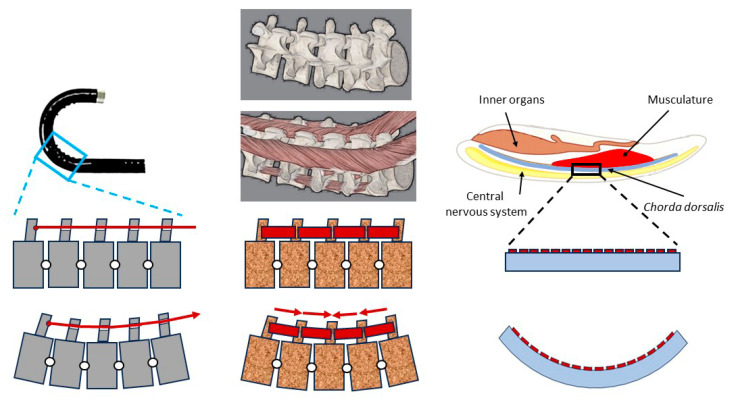
(**Left**): the principle of conventional endoscopes. (**Center**): the principle of bending a vertebrate’s spine. (**Right**): the principle of the *Chorda dorsalis* system in basic Chordates.

From a technical perspective, present endoscopes are bending beams with a rather stiff “body,” and a “neck” with underactuated rotatory DoFs (degrees of freedom) around two orthogonal bending axes each. This principle biologically roughly mirrors a motion segment [23] of a vertebral column (without the rotatory DoF around the longitudinal axis, which leads to torsion of the spine), or a mechanically coupled (not independently movable) series of motion segments. The desirable extreme to optimize the versatility and adaptability of the endoscope to changing moving paths is a continuum, which is actuated locally leading to a DoF of theoretically up to infinity. The more obvious biological paragon, which realizes this principle nearly perfectly, is obvious: the elephant’s trunk. Structures like that are mesoscale or microscale, and at present are not realizable. Biomimetics in its technical part for present customer demands must find technologically realizable solutions, and biomimetics is no one-way system from biology to engineering. One must first present a realizable step in the wanted direction for endo devices, possibly the (phylogenetically back-) step from the structure of the vertebral column (which very roughly transferred underlies the present constructions) to that of its historical precursor *Chorda dorsalis* as a biomimetic paragon, as illustrated in Figure 2. 

Figure 2, left, represents the working principle of conventional endoscopes. Bending is realized by a series of rigid bodies (forming “segments”), rotating around defined joint axes. The motion of all segments is coupled by the application of the same force via a Bowden drive. The principle of bending a vertebrate’s spine is represented in the center. This is comparable to the principle used in conventional endoscopes, with the exception that rotation in all segments may be different since forces are provided by intersegmental muscles. In the real animal, the rotation axis is slightly moving during motion, and additional longer muscles allow controlled coupled bending as well.

On the right, the principle of the *Chorda dorsalis* system is illustrated. The support structure is a compliant beam (in the natural paragon with changing geometry over length), which due to the action of small muscles can be bent locally with different curvatures along the length. For reasons of clarity, motion is shown around one axis in one direction; in technology, as in nature, actuation in up to three rotational DoFs in an antagonistic manner may provide six overlaid rotational directions. While the spine may be seen as a series of rigid segments, connected by “real” joints (diarthroses), actively moved by muscles bridging the gaps, *Chorda dorsalis* form a continuous bending beam, to which local bending is imprinted by local muscle fibers. These allow the controlled application of bending moments and axial (compressive) forces.

Transverse forces in a bending beam are coupled in a fixed manner to the bending moment around the transversal axis, thus they may also be controlled in a coupled manner as one DoF. The chorda is by no means a faulty construction; it has fulfilled its tasks for half a billion years and still fulfills them in recent animals. Beneath other factors, growing body masses and thus gravitational and inertial forces in combination with the utilization of torsional movements led to the evolution of the spinal column, which we as humans anthropocentrically consider to be the better solution. However, both the chorda and the spine are adapted to their respective tasks, and the chorda, with its lower load-bearing capacity but higher local bending, due to flexibility in combination with a monolithic structure when torsion is not required, corresponds better in its functional description to the requirements of an endoscope than a spine, so it is the more suitable biological paragon.

Derived from that (simplified) model of a chorda, we realized a demonstrator in mesoscale with a very limited number of synergistic “muscle pairs” on a continuous flexible beam (due to the lack of a yet not industrialized precise process presently feasible), which was formed by segments (technically in modular design), which are rigidly coupled to resemble a monolithic structure.

### 2.3. Proposed Soft Mechanism

#### 2.3.1. Design and Prototyping

**Figure 3:**
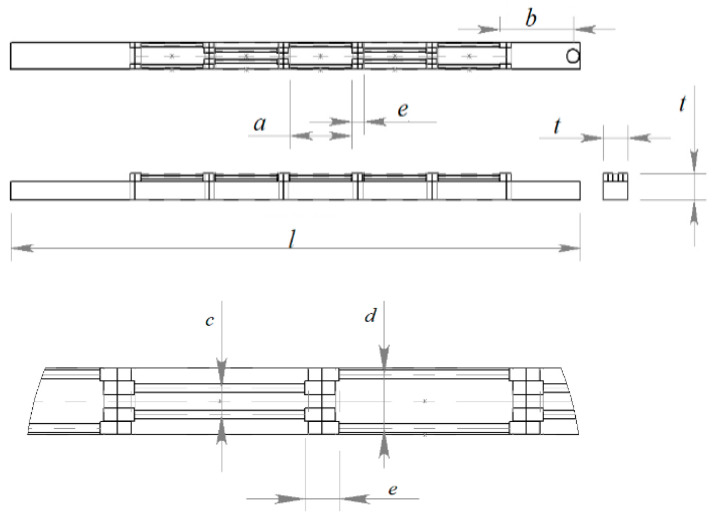
Design of the soft mechanism.

Dominantly, soft pneumatic actuators contain silicon rubbers as their main material, and generally make use of a sort of asymmetry, either via an asymmetric cross section or strengthening fibers, to yield a directed motion when pressurized air is exerted. A McKibben actuator is a type of pneumatic muscle that is attractive for biomedical applications [24], with advantages of similarity with biological muscles, reliable safety, and good performance. The actuator consists of a hollow cylinder soft tube, and a braided reinforcing net [25]. A method of fabrication of small-size McKibben actuators was proposed in [26]. Using a braider machine, like the machines used for producing braided strings, as described in [27], the actuators are manufactured in long lengths that can be cut to the desired size. In this study, we proposed a multi-segment McKibben-based soft probe, schematically shown in Figure 3. 

**Figure 4:**
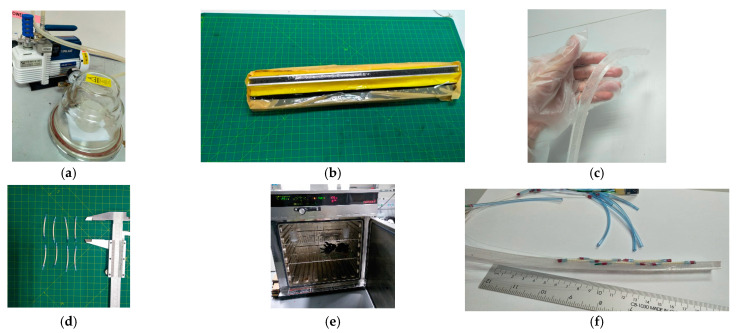
Prototyping the soft robot: (**a**) vacuum of mixed material; (**b**) molding; (**c**) trimming; (**d**) preparation of the muscles and pipes; (**e**) adhesive curing; (**f**) the fabricated prototype.

**Figure 5:**
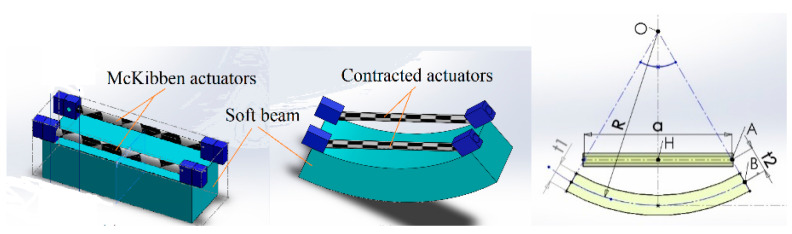
Schematic representation of segmental bending.

Each segment contains symmetrically to the midline two “muscles” or McKibben actuators of 1.3 mm diameter with equal lengths, *a* = 25 mm. Table 1 represents the dimensions. Each actuator is connected to a pneumatic pressure source and controlled individually. To investigate the feasibility of the proposed design, a prototype of the soft robot was fabricated. The main steps for prototyping are illustrated in Figure 4. For mechanical modeling purposes, we consider a single segment of the soft robot as in Figure 5. Due to compliance, the resistance force of the soft beam (bending stiffness) is negligible. This means that the external load on the McKibben actuators is zero, and their length depends on the pneumatic pressure. Thus, the quasi-static geometry relations and equations are represented as a function of the length of the actuators.

**Figure 6:**
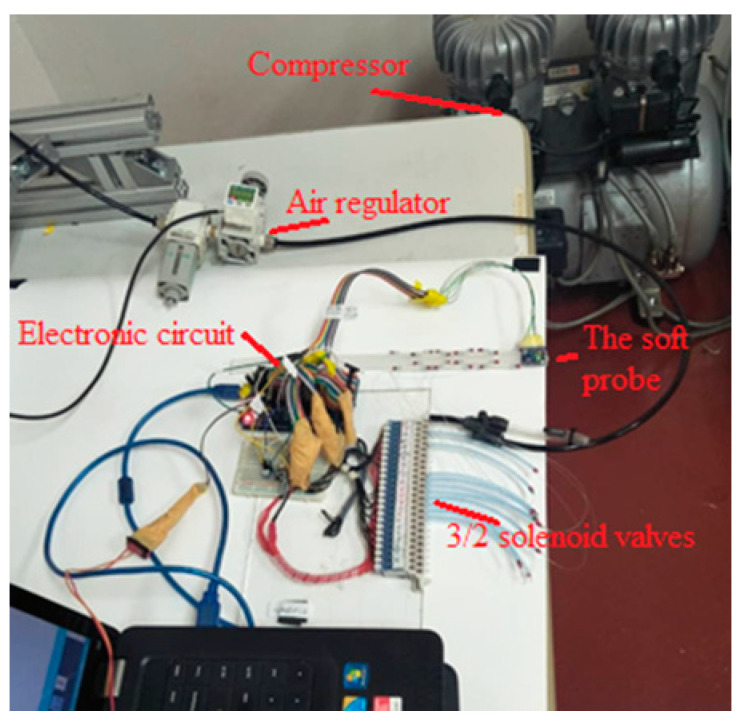
Final hardware setup.

First, the material of the core was mixed and processed by a vacuum pump for approximately one hour to reduce bubbles. The material was cast in an aluminum mold. Then, the McKibben actuators were connected to 0.5 mm pipes, and attached to the core. For more robust attachment, the same mixture of the material was used as the adhesive and was cured in an oven. The electronic hardware for the control of valves includes an Arduino^®^ Mega and the interfacing circuits (using TBD62183AFNG). The pneumatic control system includes 10 miniature 3/2 NC Solenoid valves working with 24 V. The actual system is shown in Figure 6.

The valve control system was programmed with MATLAB^®^ and deployed on Arduino^®^. An MPU 6050 as the tilt sensor was added to the system at the tip of the robot. It was programmed just to measure angles, although the sensor has more capabilities like acceleration measurement. The McKibben muscles can be pressurized individually.

#### 2.3.2. Modelization

Suppose the actuator with initial length, $a_{0}$, is contracted to $a<a_{0}$ resulting in the bending of the soft beam. Let be $\tau =t_{1}/2+t_{2}$. Then for angle θ=∡AOH, we have

(5) a=2(R−τ)Sin(θ)

Defining the contraction rate, $\lambda =a/a_{0}$, for the actuator is

(6) a=2λθR

Combining Equations (5) and (6), we obtain

(7) $$2\tau \theta \mathrm{Sin}(\theta )+\lambda a_{0}\theta -a_{0}\mathrm{Sin}(\theta )=0$$

The nonlinear Equation (7) can be solved for θ. An approximation can be written in series form as follows:

(8) $$\frac{2\tau }{5!}\theta^{6}-\frac{a_{0}}{5!}\theta^{5}-\frac{2\tau }{3!}\theta^{4}+\frac{a_{0}}{3!}\theta^{3}+2\tau \theta^{2}+(\lambda -a_{0})\theta =0+O(\theta^{7})$$

**Figure 7:**
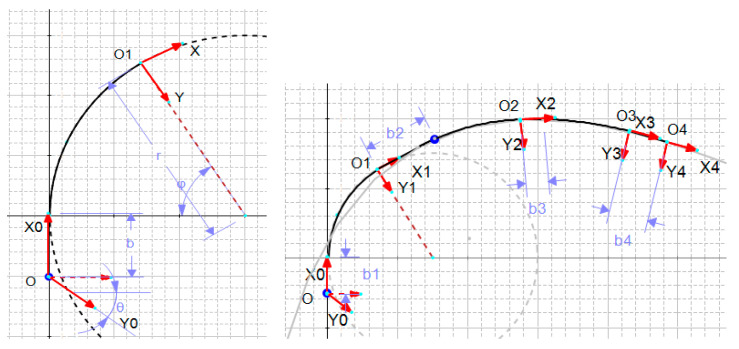
The coordinates and parameters: (**Left**): for a single segment; (**Right**): for the whole robot.

The length of the soft probe with three actuators is approximately equal to the TEE active part (and the two distal actuators are integrated for future use such as locomotion). For the three-segment model, the transformation matrix can be obtained by multiplying the transformation matrices of the segments, which in turn can be obtained in various ways. In this work, considering Figure 7, the coordinate system located at the tip is obtained by the following rotations and transformations of the base coordinate system. First, a rotation of $\theta_{x}$ around the current X-axis; a transformation along the new X- and Y-axes to reach the tip point, O_1_; and finally, a rotation of $\theta_{z}$ around the Z-axis. In case the segment is not actuated (i.e., when the pressure is zero), the transformation matrix is given merely by a transformation along the X-axis. The transformation matrix for segment 1 is represented by the double statement as follows:

(9) $$T_{1}=\left\{\begin{array}{l} Rotx(\theta_{x1})Trans(b_{1},0,0)\times \\ \;\;Trans(r_{1}.\sin(\theta_{z1}),r_{1}.[1-\cos(\theta_{z1})],0)Rotz(\theta_{z1}),\;\left(\mathrm{actuated}\right)\\ Trans(a_{01},0,0)\;\;\;(\mathrm{not}\;\mathrm{actuated}) \end{array}\right.$$

Similarly, for segments 2 and 3, it follows that

(10) $$T_{2}=\left\{\begin{array}{l} Rotx(\theta_{x2})Trans(b_{2},0,0)\times \\ \;\;Trans(r_{2}.\sin(\theta_{z2}),r_{2}.[1-\cos(\theta_{z2})],0)Rotz(\theta_{z2}),\\ Trans(a_{02},0,0) \end{array}\right.$$

(11) $$T_{3}=\left\{\begin{array}{l} Rotx(\theta_{x3})Trans(b_{3},0,0)\times \\ \;\;Trans(r_{3}.\sin(\theta_{z3}),r_{3}.[1-\cos(\theta_{z3})],0)Rotz(\theta_{z3}),\\ Trans(a_{03},0,0) \end{array}\right.$$

Note that $a_{01}=a_{02}=a_{03}=a_{0}$ and $a_{0i}=r_{i}\theta_{zi}$. For the inactive part at the tip, the transformation matrix is

(12) $$T_{4}=Trans(b_{4},0,0)$$

Finally, with the homogeneous transformation matrix *H*, the tip position in frame X_0_Y_0_Z_0_, denoted as ${\vec{P}}_{TipS}^{0}$, can be obtained related to its local coordinates as

(13) $$H_{1\to 4}=T_{1}T_{2}T_{3}T_{4}\begin{array}{ccc} & , & \end{array}{\vec{P}}_{TipS}^{0}=H_{1\to 4}{\vec{P}}_{Tip}^{4}$$

**Figure 8:**
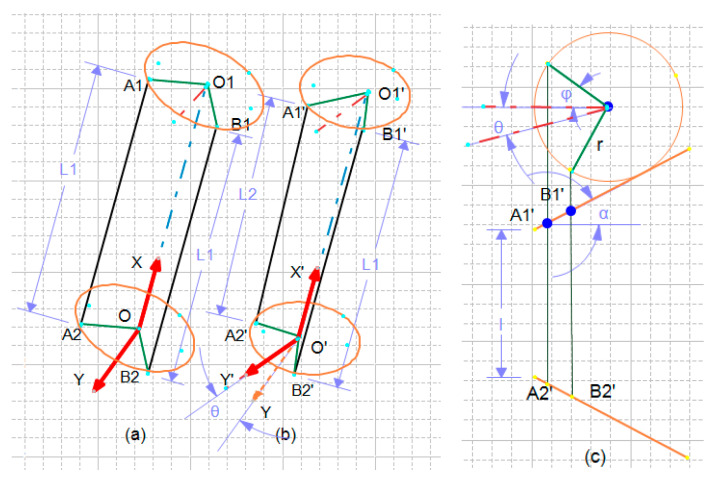
Rotation of the bending plate: (**a**) equal contraction; (**b**) asymmetric bending; (**c**) the geometry.

The application of actuators in pairs makes it possible to rotate the bending plate, and so the term *Rotx(.)* appeared based on the following description. When the actuators at both sides are equally contracted, the probe will show retroflex without lateral bending. However, if any pair of the adjacent actuators are operated with different pressures, the bending plate will rotate along X-axis as shown in Figure 8. 

As long as the length of actuators denoted as *A*_1_*A*_2_ and *B*_1_*B*_2_ are equal to *L*_1_, the *Rotx(.)* term in the equations is zero. Otherwise,

(14) L=l+2sin(α).r.(1−cos(φ))

$$L_{1}=l+2\sin(\alpha ).r.(1-\cos(\varphi_{1}))$$

(15) $$L_{2}=l+2\sin(\alpha ).r.(1-\cos(\varphi_{2}))$$

(16) $$L_{1}-L_{2}=2\sin(\alpha ).r.(\cos(\varphi_{2})-\cos(\varphi_{1}))$$

Then, the rotation angle is obtained as follows:

(17) $$\theta_{x}=\theta =-0.5(\varphi_{2}-\varphi_{1})=-0.5\mathrm{Arccos}[(L_{1}-L_{2})/2\sin(\alpha ).r]$$

The chosen state vector includes states of each segment that is the segmental bending angles. The soft probe is described with the following states:

$${\vec{q}}_{S}={\left[\theta_{x1},\theta_{z1},\theta_{x2},\theta_{z2},\theta_{x3},\theta_{z3}\right]}^{T}$$

(18) $$Q^{SoRo}=\left\{{\vec{q}}_{S}\left|0<\theta_{xi}<20^{{}^{\circ}},0<\theta_{zi}<50^{{}^{\circ}}\right.\right\}$$

For the simulations, the parameters given in Table 2 are used. Note that, in comparison with the conventional probes, the soft robot has a fundamentally different structure and mechanics. While the conventional probe is a two-DoF system with a 3D manifold workspace, the soft robot has six DoFs due to the six actuators on the active part. In fact, actuator redundancy is common in soft robots, but in most cases, the actuators (e.g., chambers in multi-chamber robots) share a single pressurizing tube. 

#### 2.3.3. Control Method

**Figure 9:**
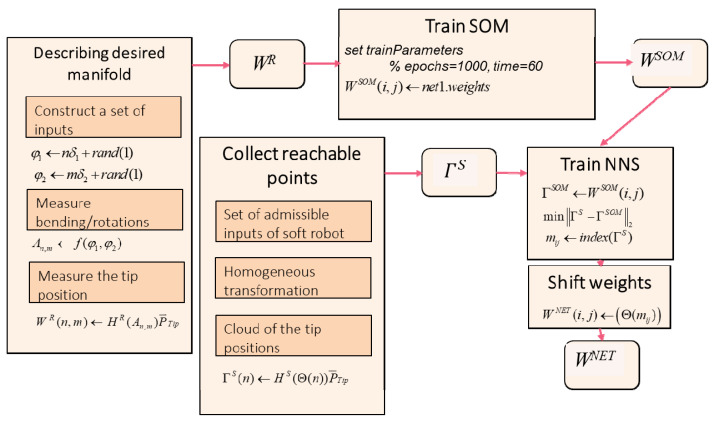
The training procedure.

Making use of the advances in data science, neural networks (NNs) are extensively used for the modeling and control of soft robots [28,29,30] to alleviate the complexity of mathematical modeling. However, the literature mainly deals with the modeling or control of soft robots as individual or independent devices like conventional robots. In this section, an algorithm is proposed to provide kinematic similarity between a reference system and a soft robot. We propose an NN based on the Kohonen (also known as SOM) network [31] and a nearest neighbor search (NNS), to provide the required kinematic similarity. The method is an unsupervised machine-learning technique that represents a two-dimensional map of the data set. Similar tools such as the k-means and related algorithms operate without topology preservation. The training is based on competitive learning. First, the distance of the input data *W^R^* to all current weight vectors *W^SOM^* is measured. Then, the neuron with the most similarity to the weight vector is labeled as the best matching unit (BMU). One single neuron is assigned as the *winning* neuron for the input points. Then, neighbor neurons in the grid are moved toward the input points. For any node *j*, the number of data points, having the node as their BMU, is calculated and assigned as *n_j_*. Then the weights are updated as a function of the average of the distances of the *n_j_* points from the node *j*, denoted as ${\bar{x}}_{j}$ with the following rule:

(19) $$W^{SOM}{|}_{node\;i}=\frac{\sum n_{j}h_{j,i}{\bar{x}}_{j}}{\sum n_{j}h_{j,i}}$$

where *h_j,i_* denotes a neighborhood distance of winning node *j* to node *i* to spread the grid over the data set. The next task of the algorithm includes an NNS, which is a kind of proximity search. The points in the cloud of soft probe tip positions closest to the SOM nodes are labeled, and the corresponding states are recorded by the algorithm. The training algorithm, which contains the offline calculations, is given as Algorithm A1 (given in Appendix A) and is represented as the block diagram in Figure 9. 

In the training algorithm, $\varphi_{1},\varphi_{2}$ can be seen as inputs of the reference system, $\delta_{1}=(\varphi_{1}^{\max}-\varphi_{1}^{\min})/N$, $\delta_{2}=(\varphi_{2}^{\max}-\varphi_{2}^{\min})/M$, and N,M∈ℕ. The set of admissible configuration angles is denoted as *A*. The subset $W^{R}\subset {\mathbb{R}}^{3}$ represents the workspace of the rigid system $\left|W^{R}\right|=NM$. The homogeneous transformation matrix is shown as $H^{R}$ and the tip position vector in the relevant local coordinate system is $\Gamma^{R}$. The SOM network is constructed with a rectangular grid of size $N_{SOM}\times M_{SOM}$ and initial weights *W^(PCA)^*. The trained SOM weight matrix is represented as $W^{SOM}$. For the soft robot, the state vector is represented as Θ, and the set of all admissible states is shown by *B*. The soft robot tip points are obtained with the soft robot transformation matrix, $H^{S}$, for all the *n* members of *B*.

For each $\Gamma^{SOM}(i,j)\in W^{SOM}$ and all $\Gamma^{S}(n)$, the index, *m_ij_*_,_ is found so that

(20) $$\begin{array}{c} {\left\|\Gamma^{S}(m_{ij})-\Gamma^{SOM}(i,j)\right\|}_{2}\leq \end{array}{\left\|\Gamma^{S}(n)-\Gamma^{SOM}(i,j)\right\|}_{2}$$

Finally, the modified network weights are saved as $W^{NET}$. The NN maps the reference input to the follower input so that the outputs meet at the representative points. Algorithm A1 is used for constructing and training the NN. Algorithm A2 (given in Appendix B) represents the way the trained system is implemented for control. In this paper, the novel algorithm that is obtained by a combination of SOM and NNS is for the first time introduced for workspace matching of robots.

## 3. Results

**Figure 10:**
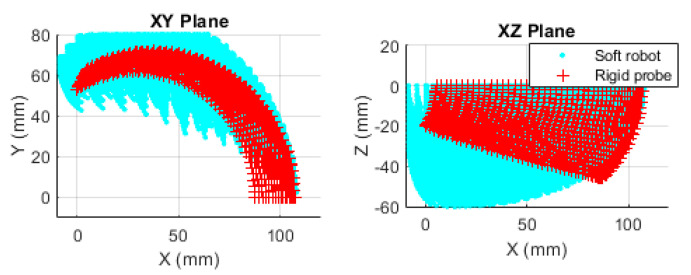
Representation of the tip access points for the soft robot and the rigid probe.

The numerical values of the tip position over all the conceivable gestures are calculated using MATLAB^®^. Figure 10 shows the results for the soft and rigid probes. The cloud of points is used to train or design the workspace. The graph necessarily shows that the soft robot can localize the US sensor to any point that is reachable by the rigid probe. This is expected from the scale and geometric similarity, but there is no point-to-point relation between the sets. The data set has a discrete and random nature, alternatively could be collected from embodied systems, and the NN is the best tool to represent such data uniformly. In this stage, two questions arise. The first regarding the feasibility of practical fabrication of the soft robot, and the second regarding the method by which such a system can be controlled. The next subsections deal with the investigation relevant to the questions. 

### 3.1. Experiments and Testing

**Figure 11:**
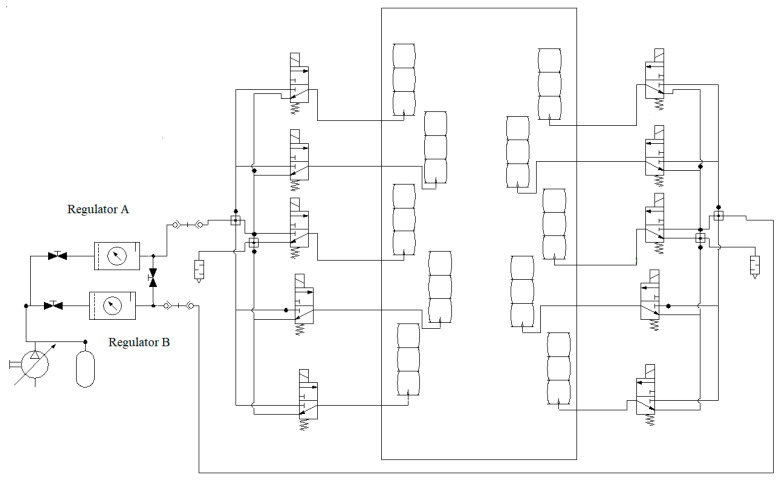
The pneumatic circuit.

**Figure 12:**
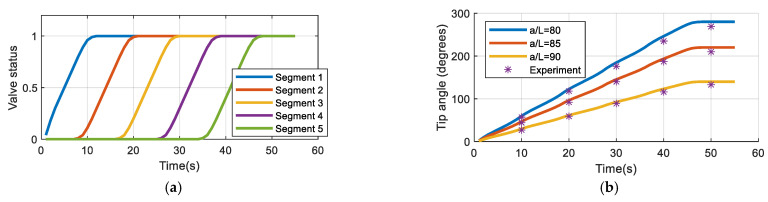
Experiment of the prototype: (**a**) the input; (**b**) the response.

**Figure 13:**
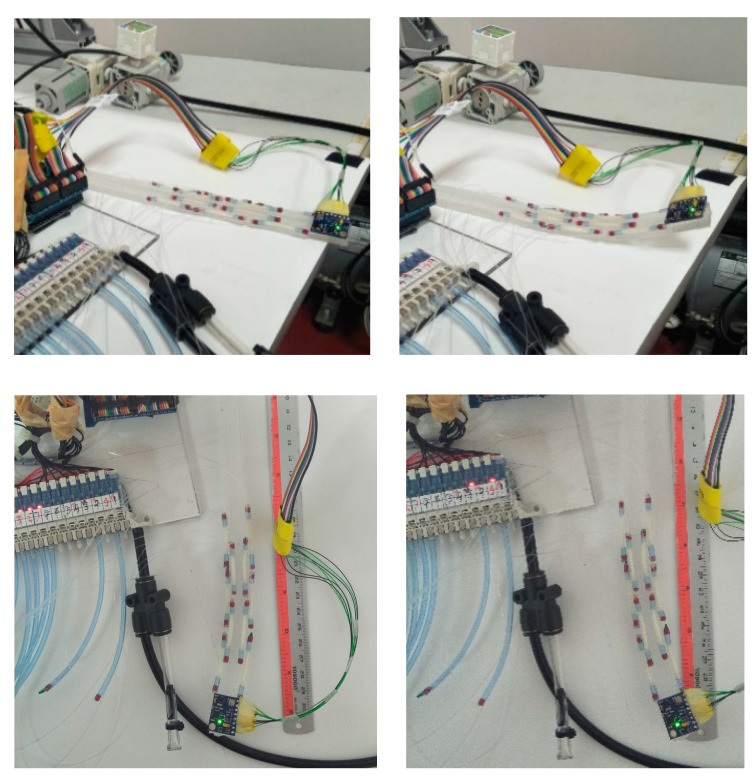
Bending to different directions.

Experimental verification was performed to evaluate the prototype as the demonstrator. For this purpose, the pneumatic circuit as shown in Figure 11 was used with an input signal that consequently (with 10 s intervals) opens the valves of each segment. The test was performed for three contraction ratios *a*/*L* (*L* = *a*_0_). The measured values are the tip angles. For a smooth transition, each pneumatic valve was excited with a PWM signal. However, only at the final states were the values read. The results are summarized in Table 3 and Figure 12. A calibration may be used to make up for the errors. Nevertheless, the small errors in this work are ignored. Note that all five segments were used in this test, so the transformation matrices of the other segments were included. Employing the standard pneumatic equipment enables us to make use of established pneumatics and commercially available components. In this work, miniature 3/2 solenoid valves with a spring return were used. Proper excitation combination of the valves provides different gestures in the robot as shown in Figure 13. Nevertheless, to have control over the pressure, more complex proportional valves are suggested. 

For this experiment, we used an open-loop control program uploaded in the Arduino^®^ Mega microcontroller. The pneumatic circuit contains miniature valves that basically have two nominal states, and are known as on/off valves. Commercially, such miniature valves are inexpensive with respect to more controllable valves. In this test, the pressure was adjusted using the manual valves on the regulators to obtain the three contraction rates. The input, that is the desired voltage applied on the valves, is increased from zero to the nominal voltage of the valves (24 V) using a PWM signal. Precise control of the pressures needs specific pneumatic valves that are generally expensive. However, with the demonstrator, the transients are not of importance and the pressure can be set to different values manually, and the valves can be controlled with the microcontroller. For example, if the lateral valves are on, the robot bends to the side. 

### 3.2. Training Results

**Figure 14:**
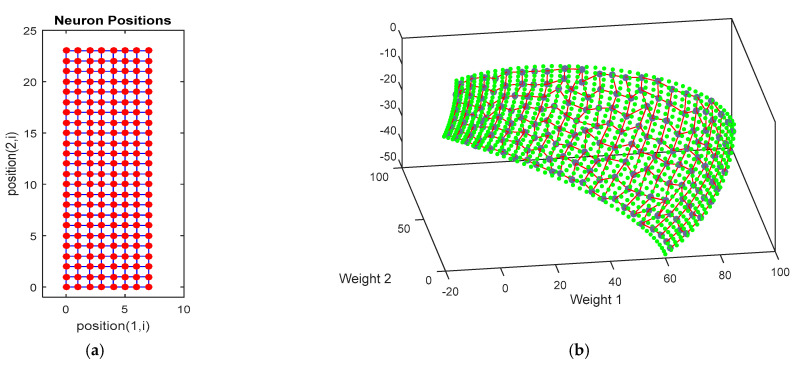
Topology of the trained network: (**a**) lattice of neurons; (**b**) the SOM weights.

Generally, the success of training depends on many factors and needs to be shown with experiments (i.e., simulations). The result’s numerical values have some randomness and can be different in different runs. Normally, repetition of training is required to achieve a good result. One advantage of the SOM nets is that the network has some visual presentation that can be used to observe the results. If the training result is not sufficiently good, some nodes will appear outside of the data (the desired manifold in this work), or the nodes will not spread uniformly. In that case, the training can be repeated by refining the parameters. The topology of the trained network used in this work is represented in Figure 14.

**Figure 15:**
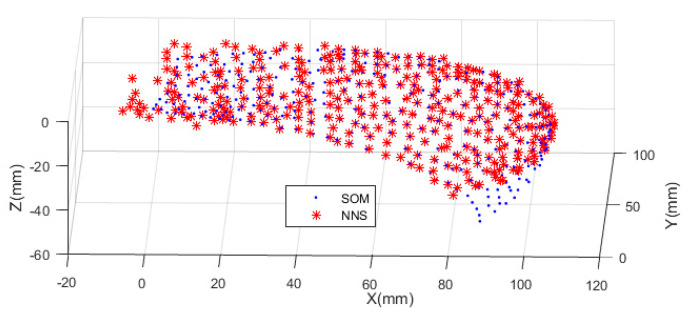
The NNS nodes versus the SOM weights.

The neurons are represented as a square lattice as in Figure 14a, where the nodes represent neurons that are loaded with weights shown in Figure 14b. The result of NNS is shown in Figure 15. The worst distance from BMUs may be taken as the error, but such an index is used in the algorithm to stop the training when the learning is not further improved. In this work, the distance between the tip of the soft robot with that of the rigid probe model is considered as the error. 

**Figure 16:**
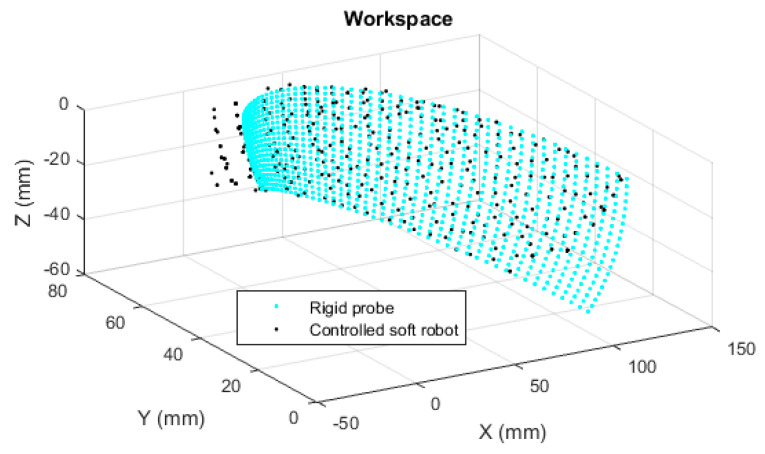
Comparison of the workspaces.

**Figure 17:**
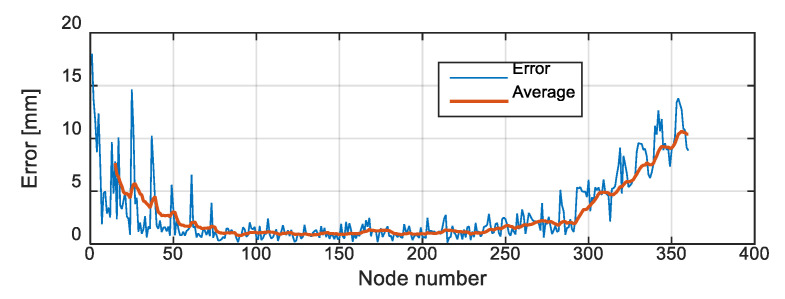
The corresponding error.

The error may be examined over the whole workspace. The NN algorithm was used to map the representative points of the workspace of the rigid probe, to the workspace of the controlled soft robot (as in Figure 16). The error was calculated by the moving average represented in Figure 17, using MATLAB^®^ syntax *avErr = tsmovavg(sqrt(e),‘s’,15,2)*;

*minimumDist* = 0.143 mm

*maximumDist* = 18.0 mm

*RMSE* = 4.36 mm

The main hyperparameters used in the training and simulation are given in Table 4. Note that the parameters can be varied depending on the application and computation power of the processor. In any case, due to randomness, one can run the training multiple times and save one trained network as the controller.

### 3.3. Case-Study Simulations

**Figure 18:**
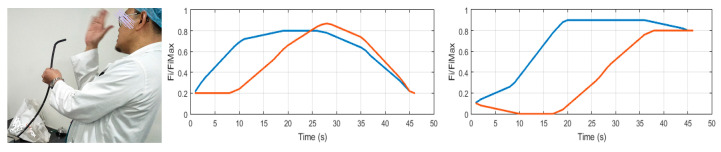
Description of the example scenarios.

**Figure 19:**
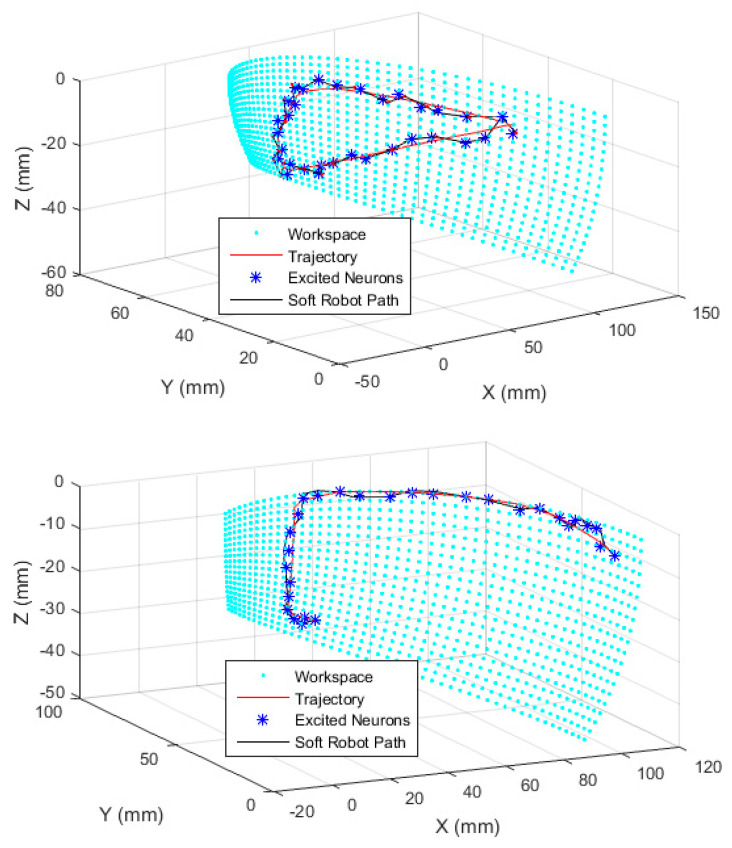
The simulation results.

**Figure 20:**
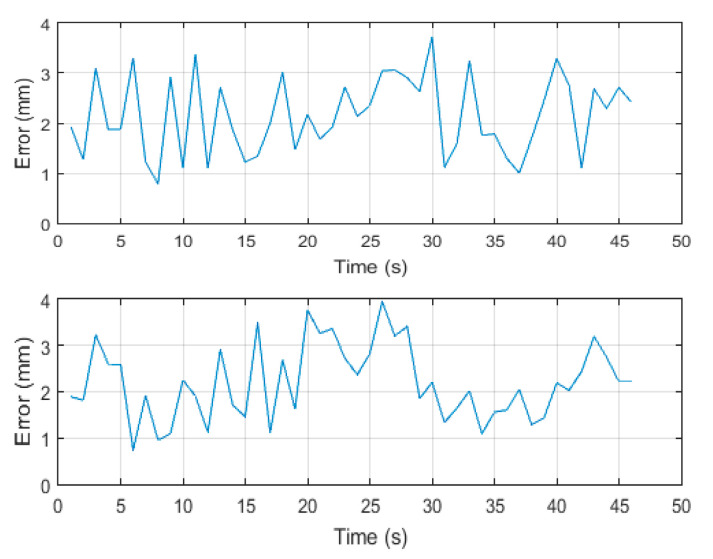
The trajectory errors.

To examine the method in the trajectory following scenarios, two simulations are presented as case studies. Note that the examples presented here are essentially arbitrary, but we had some clues from medical experts regarding practical scenarios and the normalized flexing and bending during the scenarios were reconstructed as Figure 18 and used in the simulation. In Figure 19, the desired trajectories are shown by the red line on the reference workspace manifold. The stars represent the weight of excited neurons, and the robot’s path is shown by the black line. The errors are shown in Figure 20 consequently. 

## 4. Discussion

In this work, we proposed a soft robot based on McKibben actuators that work with standard ranges of industrial pneumatic pressures (up to 600 kPa). A prototype as the demonstrator was made with equipment, components, and materials commonly accessible in academic soft robotics laboratories that have experience with soft pneumatic systems. The demonstrator was made for verification of fabrication feasibility, the model, and the bending capability of the SCR. For verification, the maximum bending of the rigid probe should be achieved by the SCR. Additionally, an arbitrary simulation of the model and experimental values obtained from the demonstrator are suggested for the verification of the model, as well as the physical system. Next, the medical requirement was retaining a kinematic similarity with the conventional system as the reference. This goal is partly achieved by designing the robot with the scale of the distal end of the probe. Furthermore, point-to-point access to the workspace corresponding to the conventional system is necessary. The SOM-based controller was designed for this aim and verified by simulations. For training, workspace points were used as the data cloud. Such data can be collected experimentally as long as a tunable pressure is available for each actuator. Note that in a TEE scenario, the sonographers first adjust the location of the US sensor at the tip of the probe using the knobs, based on their experience, and then tune adjustment of the US image is performed electronically. The medical probes used in US imaging are not moved fast and so can be described by quasi-static models, and with a kinematic matching, a soft robot will behave like the rigid counterpart without needing excessive training for the sonographer. 

A resolution (the distance between the tip of the soft robot with the desired position) within the range of 5 mm was targeted based on the recommendations of our medical advisors. It is denoted as resolution (or error) because the proposed algorithm can be retrained with different numbers of neurons to achieve different resolutions. In feedforward neural networks, an error number is calculated that can be used to indicate the training result. However, in the unsupervised NN, the error cannot be calculated by normal means. In fact, unsupervised learning has not defined error because there is no ideal data to calculate the error against it. Many SOM implementations do not even report an error and the literature does not provide an error calculation method. If higher numbers of neurons are used, the resolution can be finer at the expense of the complexity of the control system. Basically, SOMs mimic the self-organizing capabilities observed in the brain’s neural networks. The network can be trained with experimental data as well, and continuity of the soft robot input is not necessary. The results show the capability of the system to satisfy the requirements and make the method recommendable for similar applications. In the simulation results, the *RMSE* value over the whole workspace is less than 5 mm. However, the maximum value shows a large error. The error can be reduced, for example, by increasing the resolution of the SOM lattice or the number of neurons, which in turn increases the size of weight matrices, complexity, and calculation time (and delay). However, the large errors appear mainly at the ends of the workspace where the probe is either close to the straight initial status or bent to maximum angles. These states are not important because they have no contribution to US imaging. 

As future work, this study can be extended to develop the hardware, including the pneumatic pressure control system. The demonstrator shows the feasibility of the realization of the soft part and its ability to obtain the required gestures. To implement the algorithm on the soft robot, each actuator needs a pressure control system. Industrial pneumatics have commercially available solutions for this engineering practice. Moreover, as mentioned in the Introduction, researchers can develop soft pressure or flow control valves to alleviate dependence on industrial pneumatics. The prospective system can be used to provide the training data experimentally. Further research, for the specific application, includes developing a vacuum-driven SCR, which is basically safer due to negative pressure but the maneuvering and lateral bending appear more complex.

## 5. Conclusions

In this paper, a biomimetics approach was tracked for developing soft robotic probes as the next generation of conventional multilink continuum robots or transoral end effectors. The concept was that conventional endoscopes can be redesigned based on the *Chorda dorsalis* system in basic Chordates. The specific application is transesophageal echocardiography (TEE). A pneumatic soft continuum robot (SCR) was designed and fabricated integrating McKibben artificial muscles. The quasi-static models were derived and verified by experiments with the demonstrator. It was discussed that the workspace of the SCR covers that of the traditional probe, and is basically capable of performing the task. Furthermore, a novel SOM-based open-loop control strategy was developed to force the SCR kinematics to match with that of the rigid counterpart. Because SCRs and multilink mechanisms have different kinematics, the matching method is prone to errors. It was discussed that increasing the latent resolution can reduce the maximum error at the expense of more complexity. It is concluded that the SCR, which has the advantage of softness, can be a replacement for the rigid system with high usability due to kinematic similarity with the traditional system. The proposed method may be implemented for redesigning other surgical devices when the dexterity and experience of the user with the old system are important. The control method is also useful when the new system has actuator redundancy, as in our case. 

## Figures and Tables

**Table 1:** Dimensions of the soft probe.

Parameter	Description	Amount (mm)
*a*	Length of actuators	25 ± 1
*b*	Tip center position	27 ± 0.1
*c*	Lateral distance	5 ± 0.5
*d*	Lateral distance	8 ± 0.5
*e*	Longitudinal distance	2 ± 0.5
*t*	Core width	10 ± 0.1

**Table 2:** Numerical values of the simulation parameters.

	Parameter	Description	Amount (mm)
Soft robot	*a*_0_	Length of each segment	27
	*L*	Length of bending sections	25
	*b*_1_	Inactive part of segment 1	14
	*b*_2_, *b*_3_	Inactive part of segments 2 and 3	2
	*b*_4_	Length of inactive distal part	28
Rigid probe	*l*_1_, *l*_2_,…, *l*_7_	Length of rigid links	10
	*l*_8_	Length of the distal link	27

**Table 3:** Numerical values.

a/a_0_		Tip Angle (^o^)				
80	Simulation	59.9	122.6	184.8	246.4	280
	Experiment	57	118	176	235	269
	Error ^1^	−4.8	−3.7	−4.8	−4.6	−3.9
85	Simulation	47.1	96.4	145.2	193.6	220
	Experiment	44	92	140	187	210
	Error	−6.6	−4.6	−3.6	−3.4	−4.5
90	Simulation	30	61.3	92.4	123.2	140
	Experiment	27	59	89	116	133
	Error	−10	−3.8	−3.7	−5.8	−5.0

^1^ Error = (Experiment – Simulation)/Simulation × 100.

**Table 4:** The control parameters used in the MATLAB^®^ simulations.

Specification	Denomination	Set Values
Dimensions of the SOM lattice	$N_{SOM}\times M_{SOM}$	8 × 24
Layer topology function	*topologyFcn*	Gridtop ^1^
Neuron distance function	*distanceFcn*	Dist ^2^
Number of training steps	*coverSteps*	300
Initial neighborhood size	*initNeighbor*	10
Epochs	*Net.trainParam.epochs*	1000

^1^ To create a rectangular grid. ^2^ Referring to Euclidean distance.

## Data Availability

Data are contained within the article.

## Appendix A

**Algorithm A1:** Training

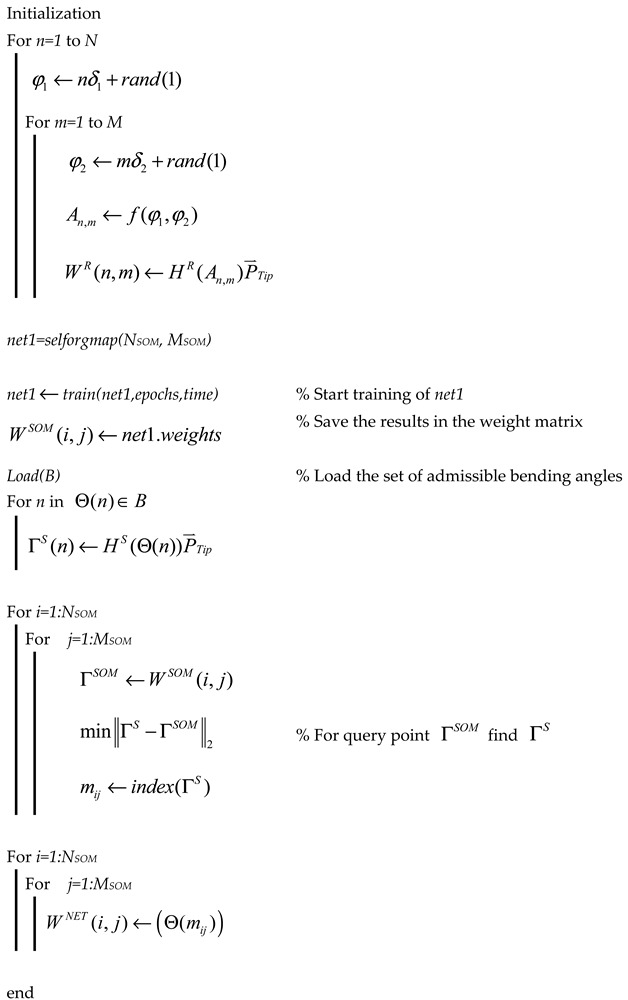

## Appendix B

**Algorithm A2:** Controller
